# Neural Synchrony Gives Rise to Amplitude- and Duration-Invariant Encoding Consistent With Perception of Natural Communication Stimuli

**DOI:** 10.3389/fnins.2020.00079

**Published:** 2020-02-05

**Authors:** Michael G. Metzen, Volker Hofmann, Maurice J. Chacron

**Affiliations:** Computational Systems Neuroscience Laboratory, Department of Physiology, McGill University, Montreal, QC, Canada

**Keywords:** invariance, weakly electric fish, identity-preserving transformations, neural coding, synchrony

## Abstract

When confronted with a highly variable environment, it remains poorly understood how neural populations encode and classify natural stimuli to give rise to appropriate and consistent behavioral responses. Here we investigated population coding of natural communication signals with different attributes (i.e., amplitude and duration) in the electrosensory system of the weakly electric fish *Apteronotus leptorhynchus*. Our results show that, while single peripheral neurons encode the detailed timecourse of different stimulus waveforms, measures of population synchrony are effectively unchanged because of coordinated increases and decreases in activity. A phenomenological mathematical model reproduced this invariance and shows that this can be explained by considering homogeneous populations whose responses are solely determined by single neuron firing properties. Moreover, recordings from downstream central neurons reveal that synchronous afferent activity is actually decoded and thus most likely transmitted to higher brain areas. Finally, we demonstrate that the associated behavioral responses at the organism level are invariant. Our results provide a mechanism by which amplitude- and duration-invariant coding of behaviorally relevant sensory input emerges across successive brain areas thereby presumably giving rise to invariant behavioral responses. Such mechanisms are likely to be found in other systems that share anatomical and functional features with the electrosensory system (e.g., auditory, visual, vestibular).

## Introduction

It is now widely accepted that behavioral responses of vertebrates to natural stimuli are determined by integrating the activities of large neuronal populations (Cohen and Kohn, 2011). However, how such integration is achieved remains poorly understood in general. This is in part because natural stimuli display complex spatiotemporal characteristics (Attias and Schreiner, 1997; Mante et al., 2005; Theunissen and Elie, 2014), as well as the fact that neuronal activities are not independent of one another (Averbeck et al., 2006). Of particular interest is the fact that neurons often fire action potentials synchronously, which is thought to enable neuronal ensembles to better encode specific stimulus features (Gray and Singer, 1989; Dan et al., 1998; Nunez and Malmierca, 2007; Uhlhaas et al., 2009; Brette, 2012; Harris and Gordon, 2015). Increased response selectivity in higher brain areas (i.e., “sparse coding”) has been observed ubiquitously (Vinje and Gallant, 2000; Laurent, 2002; Olshausen and Field, 2004; Vonderschen and Chacron, 2011; Theunissen and Elie, 2014; Sproule et al., 2015) but must be balanced by the fact that neuronal representations also become more invariant to a given sensory input encountered under different contexts (e.g., the same object under different levels of illumination) (Dicarlo and Johnson, 1999; Quiroga et al., 2005; Billimoria et al., 2008; Rust and Dicarlo, 2010; Barbour, 2011; Rust and Dicarlo, 2012; Schneider and Woolley, 2013; Sharpee et al., 2013). The mechanisms that mediate the emergence of invariant representations and the tradeoff with sparse coding remain poorly understood to this day.

Weakly electric fish generate an electric field through their electric organ discharge (EOD) and can sense perturbations through an array of electroreceptor afferents embedded in their skin (Turner et al., 1999). These afferents synapse onto pyramidal cells within the electrosensory lateral line lobe (ELL) which then project to higher brain centers that mediate behavioral responses (Rose, 2004). Natural electrosensory stimuli comprise those caused by objects such as prey (Nelson and Maciver, 1999) as well as those caused by conspecifics (Zakon et al., 2002; Metzen, 2019). In the latter case, natural electrocommunication stimuli (i.e., “chirps”) consist of transient increases in EOD frequency that occur on top of the underlying sinusoidal background beat (Zupanc and Maler, 1993; Engler et al., 2000; Bastian et al., 2001; see Zupanc, 2002 for review; Kolodziejski et al., 2005). The responses of electroreceptors and pyramidal cells to natural electrocommunication stimuli have been extensively characterized (Benda et al., 2005, 2006; Marsat et al., 2009; Marsat and Maler, 2010; Vonderschen and Chacron, 2011; Walz et al., 2014; Metzen et al., 2016; Metzen and Chacron, 2017; Allen and Marsat, 2018, 2019). In particular, for single electroreceptor afferents (EAs), it has been shown that their time-dependent firing rates will vary differentially in time when chirps with different attributes (e.g., characterized by different EOD frequency increases and/or durations) are presented on top of beats with different frequencies (Benda et al., 2005, 2006; Walz et al., 2014). At the population level, it has been shown previously that the presentation of natural electrocommunication stimuli gives rise to synchrony in the responses of EAs which is primarily seen for low beat frequencies (Benda et al., 2006; Walz et al., 2014). It is important to note that Walz et al. (2014) did not systematically vary chirp duration or the EOD frequency increase in their study. For ELL pyramidal cells (PCells), it has been previously shown that they will respond differentially to chirps with different attributes through burst firing caused in part by feedback, thus enabling better signal detection (Marsat et al., 2009; Marsat and Maler, 2010, 2012, Vonderschen and Chacron, 2011). Our previous studies have considered the coding of chirps occurring on different phases of the beat and revealed the emergence of invariant neural representations based on synchronous activity at the level of EAs (Aumentado-Armstrong et al., 2015; Metzen et al., 2016; Metzen and Chacron, 2017). However, how EAs encode chirps with different durations and amplitudes (i.e., different EOD frequency increases) has not been systematically investigated to date at either the single neuron or at the population level. Further, how this information is decoded by downstream pyramidal cells to give rise to perception and behavior has not been studied to date.

Here we used a combination of electrophysiological recordings, mathematical modeling, and behavioral assays to investigate how chirps with different amplitudes and durations are represented by peripheral electroreceptor afferent neural populations. Furthermore, we analyzed how this representation is decoded by downstream central ELL pyramidal neurons that represent a bottleneck in the electrosensory pathway and whose responses are further processed by downstream brain areas to generate electrosensory perception and behavior. Our results demonstrate that synchronous activity at the afferent population level gives rise to a representation of natural electrocommunication stimuli that is invariant to variations in stimulus attributes such as duration and amplitude. This representation is decoded by ELL PCells and these responses are further processed by downstream brain areas to generate invariant behavioral responses. Because of anatomical and functional similarities between the electrosensory and other systems (Clarke et al., 2015), the uncovered mechanism for generating invariant neuronal responses is likely to be generally applicable.

## Materials and Methods

### Ethics Statement

The animal study was reviewed and approved by McGill University’s animal care committee under protocol number 5285.

### Animals

We used a total of *N* = 20 *Apteronotus leptorhynchus* specimens of either sex in this study. Animals were acquired from tropical fish suppliers and acclimated to laboratory conditions according to published guidelines (Hitschfeld et al., 2009).

### Surgery and Recordings

Surgical procedures have been described in detail previously (Toporikova and Chacron, 2009; Vonderschen and Chacron, 2011; McGillivray et al., 2012; Deemyad et al., 2013; Metzen et al., 2016). Briefly, animals (*N* = 12) were injected with tubocurarine chloride hydrate (0.1 – 0.5 mg) for immobilization before being transferred to an experimental tank and respirated with a constant flow of water over their gills (∼10 ml/min). To expose the hindbrain for recording, a portion of the animal’s head was kept out of water and anesthetized locally with lidocaine ointment (5%). A small craniotomy (∼5 mm^2^) was made above the hindbrain for afferent and ELL PCell recordings. We used 3M KCl-filled glass micropipettes (30 MΩ resistance) to record from electroreceptor afferent axons (*N* = 60) as they enter the ELL (Savard et al., 2011; Metzen and Chacron, 2015; Metzen et al., 2015). We recorded from single EAs in response to stimulation and then recombined the activities. This is because previous studies have shown that, as EAs do not display noise correlations, similar results were obtained when considering either simultaneous or non-simultaneous recordings (Chacron et al., 2005a; Metzen et al., 2015, 2016). Extracellular recordings from ELL PCells within the lateral segment (*N* = 40) were performed with metal-filled micropipettes (Frank and Becker, 1964; Chacron et al., 2009; Chacron and Fortune, 2010; Metzen et al., 2016). The sample sizes are similar to those used in previous studies. Baseline (i.e., in the absence of stimulation) firing rates for EAs and PCells were 368 ± 113 Hz, and 12 ± 8 Hz, respectively, and were similar to previously reported values (Chacron et al., 2005b; Gussin et al., 2007; Metzen et al., 2015). We only recorded from neurons that responded to at least one chirp stimulus waveform. Recordings were digitized at 10 kHz (CED Power 1401 & Spike 2 software, Cambridge Electronic Design) and stored on a computer for subsequent analysis.

### Stimulation

The neurogenic electric organ of *A. leptorhynchus* is not affected by injection of curare-like drugs. Stimuli consisted of amplitude modulations of the animal’s own EOD and were produced by first generating a sinusoidal waveform train with frequency slightly greater (20 – 30 Hz) than the EOD frequency that was triggered by the EOD zero crossings. This train is synchronized to the animal’s EOD and will either increase or decrease the EOD amplitude based on polarity and intensity. This train is then multiplied (MT3 multiplier, Tucker Davis Technologies) with an amplitude modulated waveform (i.e., the stimulus). The resultant signal is then isolated from ground (A395 linear stimulus isolator, World Precision Instruments) and delivered to the experimental tank via two chloridized silver wire electrodes located ∼ 15 cm on each side of the animal (Bastian et al., 2002). To elicit neural and neuronal responses, we generated chirps with different attributes by systematically varying both chirp duration (8, 11, 14, 17, and 20 ms) and amplitude (10, 35, 60, 85, and 110 Hz). These ranges were chosen to contain those observed in the current study as well as those observed in previous studies (Zupanc and Maler, 1993; Engler and Zupanc, 2001; Zupanc et al., 2006). It is important to note that the chirp amplitude is not equivalent to the actual spectral frequency content of the resulting AM stimulus which is 50–100 Hz (Zupanc and Maler, 1993). Moreover, we considered chirps occurring at either phase 90° or 270° of the beat cycle, on top of a sinusoidal beat with frequency *f_*beat*_* = 4 Hz as done previously (Vonderschen and Chacron, 2011; Metzen et al., 2016). We chose a 4 Hz beat because this was the frequency used in a previous study (Metzen et al., 2016) and is characteristic of the low frequency beat stimuli encountered during interactions of two same-sex conspecifics, during which electrocommunication stimuli like those considered here occur. We chose two beat phases because our previous study has shown that EA synchrony but not single EA firing rate is invariant to different chirp waveforms with given attributes (i.e., duration and amplitude) occurring at eight different beat phases, which presumably led to invariant behavioral responses (Metzen et al., 2016). Further, we showed that ELL PCells were “locally” invariant in that they responded similarly to chirps occurring near the beat through (i.e., “+chirps”) and similarly (but in opposite fashion to “+chirps”) to chirps occurring near the beat peak (i.e., “−chirps”) (Metzen et al., 2016). The two phases chosen here correspond to representative examples of “+chirps” and “−chirps” that will effectively capture variations in neural responses due to chirps occurring at different beat phases. To measure the stimulus intensity, a small dipole was placed close to the animal’s skin. Stimulus intensity was adjusted to produce changes in EOD amplitude that were ∼20% of the baseline level, as done previously (Metzen et al., 2016; Metzen and Chacron, 2017). Finally, each chirp stimulus (i.e., a chirp with given duration and amplitude) was presented at least 20 times (i.e., 20 trials) in order to average the variability of neural responses.

### Modeling

We used the leaky integrate and fire model with dynamic threshold (LIFdt) (Chacron et al., 2000, 2001) that is an extension of the Nelson model using the following set of differential equations to account for various filtering mechanisms (Bastian, 1981; Nelson et al., 1997):

(1) $${\dot{S}}_{a}=-\frac{S_{a}}{\tau_{a}}+\frac{G_{a}}{\tau_{a}}\,A\,\left(t\right),$$

(2) $${\dot{S}}_{b}=-\frac{S_{b}}{\tau_{b}}+\frac{G_{b}}{\tau_{b}}\,A\,\left(t\right),$$

(3) $$S\,\left(t\right)=-S_{a}-S_{b}+\left(G_{a}+G_{b}+G_{c}\right)\,A\,\left(t\right),$$

where *A(t)* is the stimulus, and *S(t)* is the filtered stimulus. The *G* values are gains in units of spikes per second per millivolt (*G_*a*_* = 18300 *spikes* × s^–1^ × mV^–1^; *G_*b*_* = 850 *spikes* × s^–1^ × mV^–1^; *G*_*c*_ = 670 *spikes* × s^–1^ × mV^–1^), and the *τ* values are time constants in units of seconds (*τ_*a*_* = 0.002 s; *τ_*b*_* = 0.25 s). The total dimensionless synaptic current arriving at the spike initiation zone is given by:

(4) $$I_{\mathrm{syn}}=S\,\left(t\right)+\gamma \,A_{0}+\xi \,\left(t\right),$$

where *I*_*syn*_ is the synaptic current, *S(t)* is the filtered stimulus according to equations (1–3), γ and *A*_0_ are constants. ξ*(t)* is Gaussian white noise with zero mean and variance of one. In the time window after the absolute refractory period and up to the next action potential, the voltage *V* and the threshold θ are given by:

(5) $$\dot{V}=-\frac{V}{\tau_{\nu }}+\frac{I_{\mathrm{syn}}}{\tau_{\nu }},$$

(6) $$\dot{\theta }=\frac{\left(\theta_{0}-\theta \right)}{\tau_{\theta }},$$

where *V* is the membrane potential, τ_ν_ is the voltage decay constant of the membrane, θ is the spike threshold, and τ_θ_ is the threshold decay constant. Whenever *V* = θ, a spike occurs, and *V* is reset to zero and maintained there for the duration of the refractory period (*T*_*r*_). The threshold θ is also increased by a fixed amount Δθ and otherwise decays with time constant τ_θ_ between action potentials. Parameter values used were τ_ν_ = 1 ms; τ_θ_ = 7.75 ms; θ*_0_* = 0.08; Δθ = 0.001; *T*_*r*_ = 1 ms. Parameter values were chosen based on previous studies (Chacron et al., 2000, 2001; Savard et al., 2011) and were adjusted such that the mean firing rate of our model (392.71 ± 0.02 Hz) was within the experimentally observed range. As such, our model neurons were homogeneous and the spiking activities of the model neuron stimulated in the same way as our experimental data (i.e., same number of trials and trial length) were used to compute all measures at the single neuron level. The spiking activities of two model neurons with independent realizations of the noise ξ*(t)* were used to compute all measures at the population level.

### Analysis

All analyses were performed using custom-built routines in Matlab (The MathWorks Inc., Natick, MA, United States), these routines are freely available online at http://dx.doi.org/10.6084/m9.figshare.8041136.

### Electrophysiology

We used a total of *N* = 12 animals of either sex for electrophysiological recordings (EAs: *N* = 5; PCells: *N* = 7). ELL PCells were recorded within the lateral segment (LS) of the ELL where cells are most sensitive to high frequency communication signals (Marsat et al., 2009). This segment contains about 900 PCells, each receiving convergent input from about 1000 EAs on average (Maler, 2009). Action potential times were defined as the times at which the signal crossed a suitably chosen threshold value. From the spike time sequence, we created a binary sequence *R(t)* with binwidth Δ*t* = 0.1 ms and set the content of each bin to equal the number of spikes which fell within that bin. The time-dependent firing rates were obtained by averaging the neural or neuronal responses across repeated presentations of a given stimulus with binwidth 0.1 ms and were smoothed with a 6 ms long boxcar filter. We note that similar results were obtained when systematically varying the size of the boxcar filter between 6.25 ms and 250 ms (Figures 3, 4F, 7, 8G).

### Synchrony Between the Spiking Activities of Electrosensory Afferents

To quantify neural synchrony, we computed the cross-correlation coefficient between the spiking responses *R_*i*_(t)* and *R_*j*_(t)* of neurons *i* and *j* as was done previously (Shea-Brown et al., 2008; Metzen et al., 2015, 2016; Metzen and Chacron, 2017). As mentioned before, we randomly combined electrosensory afferents to compute synchrony, as these do not display noise correlations (Chacron et al., 2005a; Metzen et al., 2015). The time varying spiking synchrony was computed as the correlation coefficient between spike count sequences *S*_*i*_ obtained from the binary sequences for non-overlapping 5 ms bins during a time window of 31.25 ms that was translated in steps of 0.25 ms using:

(7) $$\rho =\frac{\mathrm{Cov}\,\left(S_{1},S_{2}\right)}{\sqrt{\mathrm{Var}\,\left(S_{1}\right)\,\mathrm{Var}\,\left(S_{2}\right)}}$$

Here, *Cov*(…) is the covariance while *Var*(…) denotes the variance, and *S*_1_, *S*_2_ are the spike count sequences from neurons 1 and 2, respectively. The time-dependent synchrony measures were then averaged across trials. We note that similar results were obtained when systematically varying the time window length between 6.25 ms and ∼60 ms but that synchrony values decreased for longer lengths up to 250 ms (Figures 5G, 6G).

### Quantifying Neural Response Invariance

The invariance score for either parameter (i.e., duration or amplitude) was defined as (Metzen et al., 2016; Metzen and Chacron, 2017):

(8) $$\mathrm{Invariance}=1-\frac{\sum_{i\neq j}\left[\frac{D\,\left({\mathrm{FR}}_{i}\,\left(t\right),{\mathrm{FR}}_{j}\,\left(t\right)\right)}{D\,\left(S_{i}\,\left(t\right),S_{j}\,\left(t\right)\right)}\right]}{N_{\mathrm{chirps}}\,\left(N_{\mathrm{chirps}}-1\right)},$$

where *N_*chirps*_* = 10 and the sum runs over indices *i* and *j* representing different values of the parameter (i.e., duration or amplitude) for all possible combinations of i ≠ j. *D(x,y)* is a distance metric between x and y that was computed as (Aumentado-Armstrong et al., 2015; Metzen et al., 2016; Metzen and Chacron, 2017):

(9) $$D\,\left(x,y\right)=\frac{\sqrt{\langle {\left(x-\left\langle x\right\rangle -y+\left\langle y\right\rangle \right)}^{2}}\rangle }{\max\left[\frac{\max\left(x\right)-\min\left(x\right)}{\sqrt{2}},\frac{\max\left(y\right)-\min\left(y\right)}{\sqrt{2}}\right]},$$

where < … > denotes an average over an evaluation window of 30 ms after chirp onset that is shown as a gray band in the figures, *FR_*i*_(t)* is the peri-stimulus time histogram (PSTH) response of a given cell to chirp stimulus waveform *S_*i*_(t)*, and max(…), min(…) denote the maximum and minimum values, respectively. All responses were normalized prior to computing the distance metric. We note that, according to equation (8), the distance between responses to two different stimulus waveforms is normalized by the distance between the stimulus waveforms themselves. A value of one indicates perfect invariance, whereas a value of zero indicates that a neuron whose response faithfully encodes the detailed timecourse of the different stimulus waveforms will not be considered invariant according to our definition. It is important to note that, unlike the detectability measure described below, our invariance measure is based on the timecourse of the actual neural responses and not solely on their minimum and maximum values. Thus, in order to obtain a high invariance score, it is not sufficient for different neural responses to merely have the same minimum and maximum values, they actually have to have a similar timecourse. It is furthermore important to note that the invariance score was computed from the PSTH responses which are averaged over trials to reduce variability. It is thus unlikely that the invariance scores reported in the current study are due to large response variability. Invariance scores were computed for each individual cell and subsequently averaged across the respective populations. We computed duration and amplitude invariance for synchronous activity as described above except that we used the timecourse of the varying correlation coefficient instead of spike counts as an input.

### Detectability

To determine the detectability of a stimulus waveform resulting from a chirp with a specific amplitude or duration within the ongoing beat, we computed the distance *D(x,y)* [equation (9)] between the chirp waveform and the corresponding beat waveform (i.e., the beat waveform when no chirp occurred) as done previously (Aumentado-Armstrong et al., 2015; Metzen and Chacron, 2017). A value of one indicates perfect detectability, whereas a value of zero indicates that the chirp waveform is identical to the beat waveform. The neuronal detectability of a chirp (using either single unit firing rate or synchronous activity) was computed using:

(10) $${\mathrm{Detectability}}_{\mathrm{neuronal}}=\mathrm{abs}\,\left(\frac{R_{\mathrm{chirp}}-R_{\mathrm{beat}}}{R_{\mathrm{chirp}}+R_{\mathrm{beat}}}\right),$$

where *R_*chirp*_* = *R*_*max*_ – *R*_*min*_ (i.e., the difference between the maximum and minimum values of the response). *R*_*chirp*_ was computed over a time window of 15 ms for EA firing rate and of 60 ms for EA synchrony and PCell firing to account for differences in the timecourse of responses as done previously (Metzen and Chacron, 2017). *R*_*beat*_ = *R*_*max*_ – *R*_*min*_ is the difference between the maximum and minimum values of the response (i.e., either of EA firing rate, EA synchrony, or PCell firing rate) to the undisturbed beat during one beat cycle, respectively. We note that this measure is similar to the chirp selectivity index used in previous studies (Vonderschen and Chacron, 2011; Aumentado-Armstrong et al., 2015).

### Behavior

*Apteronotus leptorhynchus* has been shown to robustly give chirp echo responses when stimulated with chirps (Hupé and Lewis, 2008; Gama Salgado and Zupanc, 2011). Measuring this chirp echo response has been effectively used before to infer the perceptual abilities of these animals under different stimulus conditions (Metzen et al., 2016; Metzen and Chacron, 2017). Moreover, chirping behavior was shown to be identical in freely moving and restrained fish (Hitschfeld et al., 2009). We therefore measured the chirp echo behavioral response by restraining fish (*N* = 8) in a “chirp chamber” as described previously (Metzen and Chacron, 2014; Metzen et al., 2016). Stimuli were delivered by two electrodes spaced 10 cm from each other located on the right side of the animal (Figure 9A, S1 and S2). The EOD was measured between electrodes placed near the head and tail, amplified (Axoclamp 2B, Molecular Devices), digitized at 10 kHz sampling rate using CED 1401plus hardware and Spike2 software (Cambridge Electronic Design), and stored on a computer hard disk for offline analysis. Previous studies have shown that stimulation with low frequency (<10 Hz) beats will induce chirping behavior but that this habituates over time (Bastian et al., 2001). As such, we initially habituated the animal to a 4 Hz beat stimulus lasting 60 s in order to minimize the probability of chirp responses being elicited due the beat alone. Computing the baseline chirp rate during the first (control) and last (habituated) 30 s of the habituation period showed a significant drop in chirp rate down to 0 (control: 0.5919 ± 1.137 chirps × s^–1^; habituated: 0 ± 0 chirps × s^–1^; paired *t*-test; *p* = 1.28 × 10^–5^), indicating that the animals were habituated to the beat signal. It is therefore highly unlikely that any echo response observed after stimulus chirp onset was due to the underlying beat as the chirp rate immediately before stimulus chirp onset was null. We then randomly interspersed chirp stimuli at variable intervals (15 s ± 3 s) and the recording was started 200 ms before chirp onset. To analyze the chirp echo response, we first extracted the time varying EOD frequency of each fish tested. Echo response chirps after stimulus chirp onset were identified as increases in the animal’s own EOD frequency that exceeded 30 Hz (Bastian et al., 2001). The time of occurrence of echo response chirps was defined as the time at which the EOD frequency excursion was maximal. The echo response chirp rate was computed as the number of echo response chirps during a time window of 1 s following the stimulus chirp onset since previous studies have shown that the majority of responses occur during this time window (Zupanc et al., 2006). Invariance scores for behavior were computed as described above for neural and neuronal responses except that we used the behavioral PSTHs computed from the echo responses using a 1 s boxcar filter as responses (Metzen et al., 2016). We note that we randomly varied the beat phase at which the chirp occurred between 0° and 315° in increments of 45° for either of the two chirp parameters (i.e., duration and amplitude) to avoid habituation.

### Chirp Statistics

In order to quantify the distribution of chirp attributes duration and amplitude in naturally occurring electrocommunication signals, we analyzed the chirps elicited by our fish population (*N* = 8) during the habituation period to a 4 Hz beat stimulus of 60 s duration. To do so, we extracted the time-varying EOD frequency by computing the inverse of the timing difference between successive zero crossings as done previously (Metzen et al., 2016). Chirp amplitude was computed as the difference between the baseline EOD amplitude and the maximal EOD frequency during a chirp event. The chirp duration was defined as the full width at half-maximum of the EOD frequency excursion. The time of occurrence of the chirp was defined as the time at which the EOD frequency is maximal (Aumentado-Armstrong et al., 2015). As mentioned above, previous studies have shown that, in response to stimulation with low frequency (<10 Hz) beats, animals will emit chirps. However, these studies have also shown that the characteristics of the emitted chirps (e.g., duration and amplitude) will depend on the stimulation protocol such as the beat frequency as well as stimulus intensity (Zupanc and Maler, 1993; Bastian et al., 2001; Gama Salgado and Zupanc, 2011). In contrast, our measurements of chirp statistics were made under the same conditions (i.e., same beat contrast and frequency) than those used to investigate neural and echo responses to these, thereby making them more directly comparable.

### Statistics

Statistical significance was assessed through a paired t-test or a one-way analysis of variance (ANOVA) with the Bonferroni method of correcting for multiple comparisons at the *p* = 0.05 level. Values are reported as boxplots unless otherwise stated. Errorbars indicate mean ± SD. On each box, the central mark indicates the median, and the bottom and top edges of the box indicate the 25th and 75th percentiles, respectively. Outliers are plotted individually using the symbol. 95% confidence intervals were estimated using the t-distribution.

## Results

**FIGURE 1:**
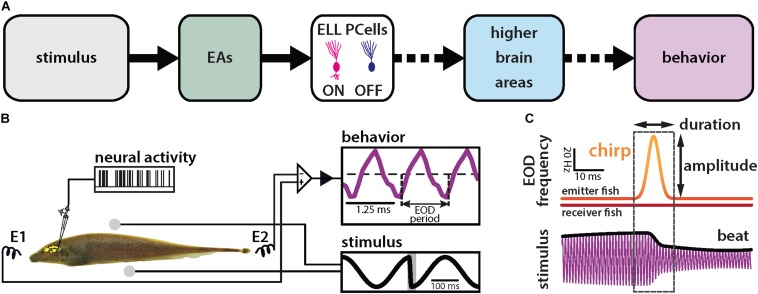
Schematic of the experimental setup. **(A)** Schematic showing successive brain areas involved in the processing of electrosensory stimuli. **(B)** Experimental setup: the animal’s electric field (i.e., the behavior, purple) is monitored by a pair of electrodes located in front and behind the animal (E1 and E2) while neural and neuronal activity is recorded. The stimulus (black) is delivered using a separate set of electrodes positioned on each side (spheres). The shaded gray rectangle in the lower right inset shows the timewindow used for analysis. **(C)** During a chirp, the emitter fish’s EOD frequency (top orange trace) is transiently increased by a maximum of amplitude for a brief duration (dashed box) while the receiver fish’s EOD frequency (red trace) remains constant. This can be characterized by the duration and amplitude of the frequency excursion. The chirp results in a phase reset of the beat (bottom black trace).

We investigated how natural electrocommunication signals (i.e., “chirps”) with different attributes are encoded by peripheral electroreceptor afferents (EAs) and their downstream target pyramidal cells (PCells) within the ELL to give rise to behavior (Figure 1A). To do so, we used an immobilized preparation in which neural, neuronal and behavioral responses can be recorded simultaneously (Figure 1B). Under natural conditions, chirps occur during social interactions in which the emitter fish sends the signal to the receiver fish. This signal consists of a transient increase in the emitter’s fish’s EOD frequency with given time duration and amplitude (i.e., the amount by which the EOD frequency increases; Figure 1C, top panel). Interactions between the two fish’s EOD frequencies gives rise to a sinusoidal background beat (Figure 1C, bottom panel, black). The chirp signal perturbs the underlying beat amplitude when considering the stimulus sensed by the receiver fish (Figure 1C, bottom panel, black).

We first investigated how chirp amplitude and duration were distributed in emitted chirps of our fish population used for behavior (*N* = 8). To do so, fish were stimulated with a background beat of 4 Hz, which is characteristic of the low frequency stimuli encountered during natural interactions between same-sex conspecifics, and the resulting chirps were detected and analyzed (see section “Materials and Methods”). While previous studies have shown that fish will emit chirps when stimulated by beats alone (Zupanc and Maler, 1993; Bastian et al., 2001; Gama Salgado and Zupanc, 2011), these have shown that the attributes of the emitted chirps (e.g., their duration and amplitude) can vary based on beat attributes such as frequency (Bastian et al., 2001). Thus, in order to ensure that our results can be directly comparable, we measured the characteristics of emitted chirps (i.e., amplitude, duration) using the same beat stimulus (i.e., same frequency and amplitude) that was used to stimulate neurons and behavioral echo responses as described below.

**FIGURE 2:**
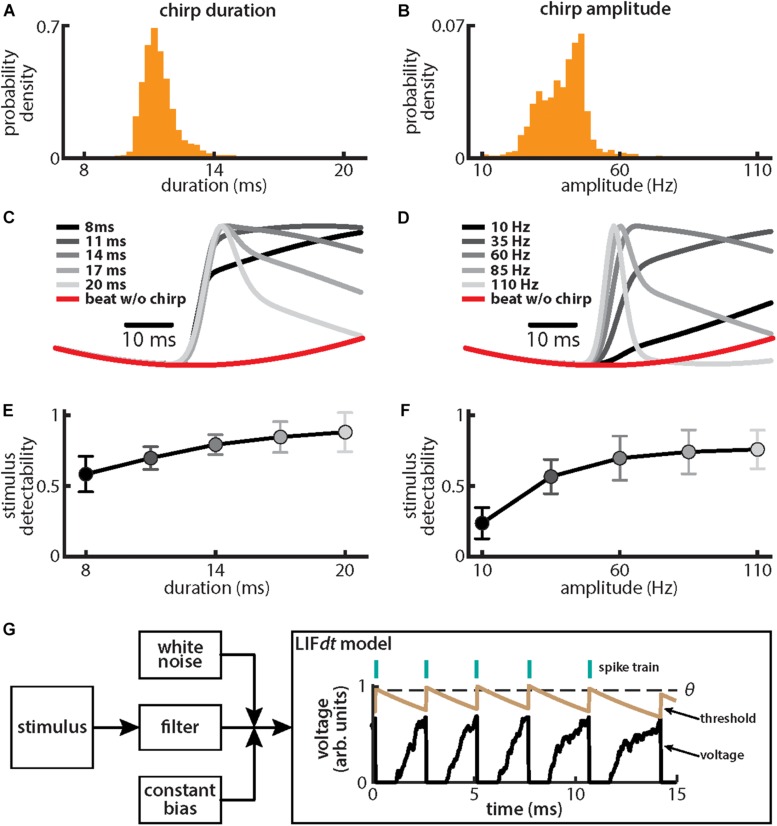
Chirps with different durations and amplitudes give rise to heterogeneous waveforms. **(A)** Probability density for chirp duration measured from chirps that were emitted by animals when stimulated with a 4 Hz beat stimulus. **(B)** Same as **(A)**, but for chirp amplitude. **(C)** Chirp waveforms for different durations, but a fixed amplitude (shaded gray). Also shown is the stimulus waveform of the beat without a chirp (red). Values are plotted as mean ± SE. **(D)** Same as **(C)**, but for chirp amplitude. **(E)** Chirp stimulus detectability as a function of duration. Values are plotted as mean ± SE. **(F)** Same as **(E)**, but for amplitude. **(G)** Schematic of the leaky integrate and fire model with dynamic threshold (LIFdt) where a stimulus is passed to a filter with and a white noise term as well as a current bias is added. The voltage (black curve) and threshold (brown curve) trace obtained with the LIFdt model showing the firing rule. When voltage becomes greater than the threshold θ, a spike is said to have occurred, and the voltage is reset to zero, whereas threshold is incremented by a constant Δθ. The threshold is kept constant to simulate the absolute refractory period *T*_*r*_ (equal to one EOD cycle) and then decays exponentially with time constant τ_θ_ to its equilibrium value θ*_0_*. Parameter values used are given in the section “Materials and Methods.”

We found that, for both duration (Figure 2A) and amplitude (Figure 2B), the distributions were relatively narrow (duration: mean: 11.45 ms, SD: 0.75 ms; amplitude: mean: 39.1 Hz, SD: 8.0 Hz). These results are consistent with previous ones (Bastian et al., 2001). We further found that the different stimulus waveforms resulting from chirps with different duration and amplitude became progressively more different from the background beat itself (Figures 2C,D), consistent with previous findings (Benda et al., 2005; Walz et al., 2014). We quantified these differences by computing stimulus detectability and found larger values for higher values of either duration (Figure 2E) or amplitude (Figure 2F). To better understand experimental results, we used a phenomenological mathematical model of EA activity that closely reproduces experimental results (see section Materials and Methods). The model consists of a leaky integrate and fire formalism with dynamic threshold (LIFdt) for spike generation to which input in the form of the filtered stimulus based on single neuron properties found experimentally (Xu et al., 1996), noise, and a constant bias are given (Figure 2G). The model afferents were simulated using independent sources of noise (see section Materials and Methods), which assumes that there are no noise correlations and is consistent with available experimental data (Chacron et al., 2005a; Metzen et al., 2016).

### Single Peripheral Afferents Respond Differentially to Natural Electrocommunication Stimuli With Different Durations and Amplitudes

**FIGURE 3:**
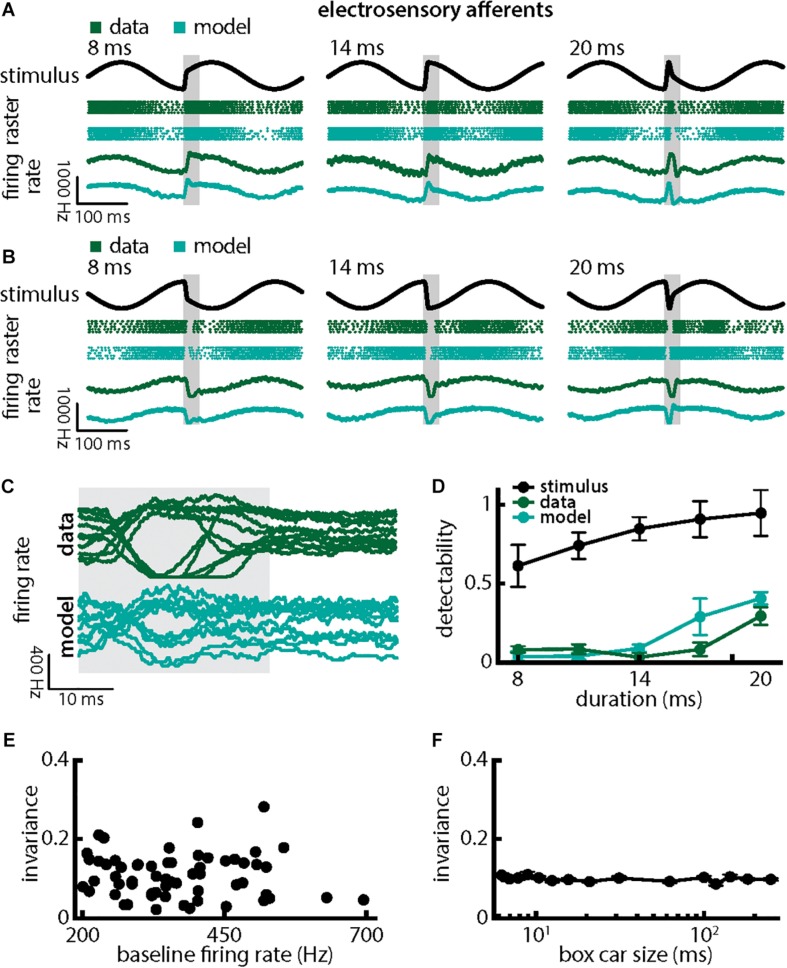
Single peripheral electroreceptor afferents respond differentially to chirps with different durations. **(A)** Example stimulus waveforms (top, black) for chirps with different durations (left: 8 ms; middle: 14 ms; right: 20 ms) occurring at the same phase of a 4 Hz beat, raster plots of an example afferent (middle top, green) and model neuron (middle bottom, cyan) showing responses to 5 out of 20 randomly chosen presentations (i.e., trials) of each stimulus and the corresponding firing rates of both neurons averaged over all 20 trials (bottom). The horizontal bars (shaded gray) represent the chirp window used for evaluation. The gray band shows the evaluation time window used to compute invariance (see below). **(B)** Same as **(A)**, but when the chirp occurred at a different phase of the beat. **(C)** Superimposed trial-averaged firing rate responses of an example afferent (green) and our model (cyan) to chirps of different durations. **(D)** Population-averaged detectability values computed from firing rate responses of the afferents (green) and the model (cyan) as a function of duration. Also shown is the stimulus detectability (black) as a function of duration. **(E)** Invariance as a function of the baseline (i.e., in the absence of stimulation but in the presence of the animal’s unmodulated EOD) firing rate for our afferent dataset. No significant correlation was observed (Pearson’s correlation coefficient, *r* = –0.0483; *p* = 0.7139). **(F)** Population-averaged invariance as a function of the boxcar low-pass filter size used to obtain the time dependent firing rate from spiking activity.

We first investigated how chirps with different durations were encoded by single EAs (Figure 3). We found that responses to these consisted of patterns of increases and decreases in firing activity that faithfully encoded the stimulus waveform (Figures 3A,B; green dots showing raster plots and green curves showing the trial-averaged firing rate), consistent with previous results (Benda et al., 2005, 2006; Walz et al., 2014). EA firing activity increased when the chirp waveform (Figure 3A, black) occurred near the beat trough (Figure 3A, green) but instead decreased when the chirp waveform occurred near the beat peak (Figure 3B, green). Superimposing the different responses emphasized differences (Figure 3C, top green). Simulations of our LIFdt model’s response to the different waveforms were in good qualitative agreement with experimental data (Figures 3A–D, cyan). Overall, stimulus detectability computed from single EA responses increased with increasing duration (Figure 3D, green, ANOVA with Bonferroni correction, *p* = 3.311 × 10^–173^, df = 295, *n* = 60) but were much lower than those computed from the stimulus (Figure 3D, black), which is due to the fact that EA responses to chirps with different durations differed from one another rather than due to variability between individual responses to a given chirp. This is because the invariance measure is computed using the trial-averaged time dependent firing rates (i.e., firing rates averaged over repeated presentations of the stimulus waveform associated with a chirp with given duration and amplitude) rather than single-trial responses which are more variable (see Section Materials and Methods). Detectability computed from our model’s responses closely matched values from experimental data (Figure 3D, compare green and cyan). Afferent heterogeneities as quantified by the baseline firing rate (i.e., in the absence of stimulation) did not affect invariance as no significant correlation was observed (Figure 3E, Pearson’s correlation coefficient, *r* = −0.0811; *p* = 0.3238). Finally, our invariance results were robust to changes in filter settings used to obtain the trial-averaged time-dependent firing rate from spiking activity (Figure 3F).

**FIGURE 4:**
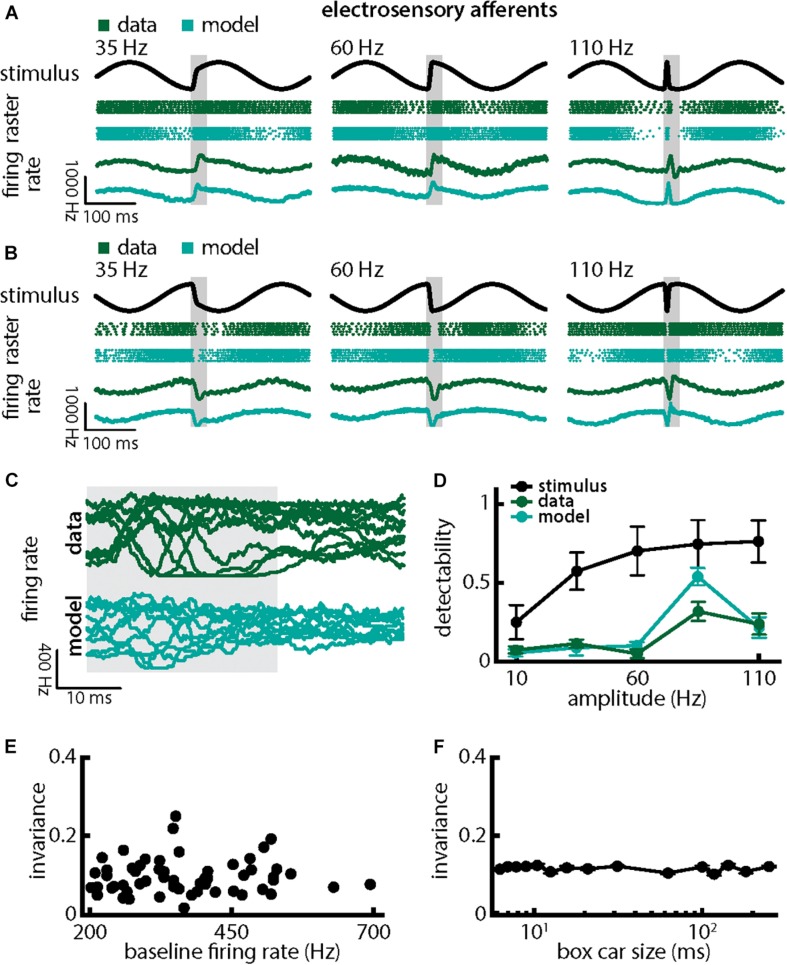
Single peripheral electroreceptor afferents respond differentially to chirps with different amplitudes. **(A)** Example stimulus waveforms (top, black) for chirps with different amplitudes (left: 35 Hz; middle: 60 Hz; right: 110 Hz) occurring at the same phase of a 4 Hz beat, raster plots of an example afferent (middle top, green) and model neuron (middle bottom, cyan) showing responses to 5 out of 20 randomly chosen presentations (i.e., trials) of each stimulus and the corresponding firing rates of both neurons averaged over all 20 trials (bottom). The horizontal bars (shaded gray) represent the chirp window used for evaluation. The gray band shows the evaluation time window used to compute invariance (see below). **(B)** Same as **(A)**, but when the chirp occurred at a different phase of the beat. **(C)** Superimposed trial-averaged firing rate responses of an example afferent (green) and our model (cyan) to chirps of different amplitudes. **(D)** Population-averaged detectability values computed from firing rate responses of the afferents (green) and the model (cyan) as a function of amplitude. Also shown is the stimulus detectability (black) as a function of amplitude. **(E)** Invariance as a function of the baseline (i.e., in the absence of stimulation but in the presence of the animal’s unmodulated EOD) firing rate for our afferent dataset. No significant correlation was observed (Pearson’s correlation coefficient, *r* = 0.0386; *p* = 0.7694). **(F)** Population-averaged invariance as a function of the boxcar size used to obtain the time dependent firing rate from spiking activity.

Qualitatively similar results were obtained when we varied chirp stimulus amplitude (Figure 4). Responses consisted of patterns of increases and decreases in firing activity that faithfully encoded the stimulus waveform (Figures 4A,B; green). Superimposing the different responses again emphasized differences (Figure 4C, top, green). Detectability also increased with increasing amplitude (Figure 4D, green curve; ANOVA with Bonferroni correction, *p* = 9.61 × 10^–133^, df = 295, *n* = 60). Results obtained from numerical simulations of our model were in good qualitative agreement with experimental data overall (Figures 4A–D, cyan). Afferent heterogeneities as quantified by the baseline (i.e., in the absence of stimulation) firing rate also did not affect invariance as no significant correlation was observed (Figure 4E, Pearson’s correlation coefficient, *r* = 0.0386; *p* = 0.7694). Finally, our invariance results were robust to changes in filter settings used to obtain the trial-averaged time-dependent firing rate from spiking activity (Figure 4F). Thus, we conclude that single peripheral afferents respond differentially to the different stimulus waveforms associated with changes in both duration and amplitude for natural electrocommunication stimuli.

### Afferent Populations Respond With Similar Increases in Synchrony to Stimuli With Different Durations and Amplitudes and Thus Provide an Invariant Representation of Both Stimulus Attributes

**FIGURE 5:**
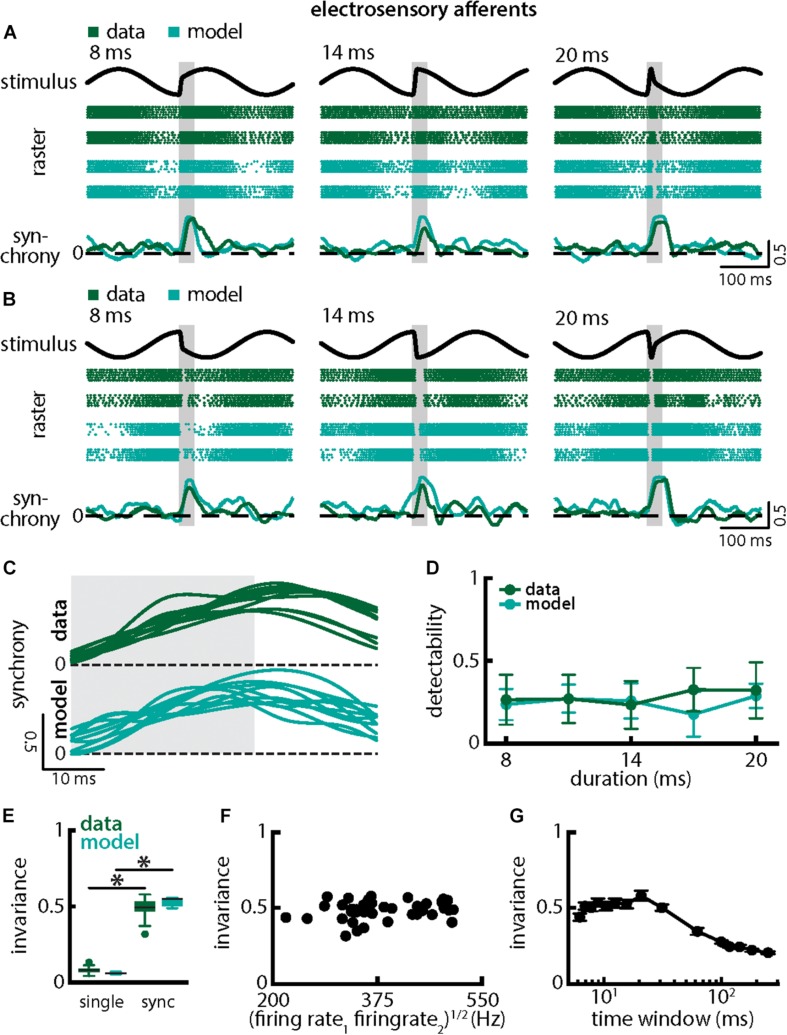
Synchrony provides an invariant representation of chirps with different durations. **(A)** Example stimulus waveforms (top) for chirps with different durations (left: 8 ms; middle: 14 ms; right: 20 ms) occurring at the same phase of the 4 Hz beat, raster plots of two example afferents (middle top, green) and model neurons (middle bottom, cyan) showing responses to 5 out of 20 randomly chosen presentations (i.e., trials) of each stimulus and the time varying synchrony averaged over all 20 trials (bottom) from the shown example afferent (green) and model (cyan) pairs. The horizontal bars (shaded gray) represent the chirp window used for evaluation. The gray band shows the evaluation time window used to compute invariance (see below). **(B)** Same as **(A)**, but when the chirp occurred at a different phase of the beat. **(C)** Superimposed trial-averaged synchrony responses from the same example pair of afferents for experimental data (green) and from a pair of model afferents (cyan) for chirps of different durations. **(D)** Population-averaged neuronal detectability values computed from the spiking synchrony from the afferents (green) and the model (cyan) as a function of duration. **(E)** Population-averaged invariance values computed for the single afferents (green) and for the model (cyan) from single afferent activity (left) and from synchrony (right) for chirp duration. “^∗^” indicates statistical significance at the *p* = 0.05 level using a paired *t*-test. **(F)** Invariance as a function of the geometric mean of the afferent baseline firing rates for our dataset. No significant correlation was observed (Pearson’s correlation coefficient; *r* = 0.1924; *p* = 0.2343). **(G)** Invariance as a function of time window length. Invariance was more or less independent of time window length for values up to ∼60 ms.

We next investigated how afferent populations encode natural electrocommunication stimuli with varying duration (Figure 5). Our results show that the spiking activities of afferent pairs were more synchronized in response to all stimulus waveforms (Figures 5A,B, green). We thus quantified the time-varying synchrony from pair-wise correlations between afferent activities which ranges between -1 (perfect anti-synchrony) and 1 (perfect synchrony) with 0 indicating lack of synchrony (see section Materials and Methods). It is important to note that the synchrony measure was averaged over trials (i.e., repeated presentations of the stimulus waveform associated with a chirp with given duration and amplitude, see section Materials and Methods) in order to ensure that changes are not due to trial-to-trial variability in the neural responses. We found that synchronous activity was much higher when a chirp had occurred than during the background beat. Synchrony transiently increases in a similar fashion in response to all chirps of different durations and irrespective of whether the stimulus occurred at the beat peak or trough (Figures 5A,B, bottom panels, 5C, top green). Overall, synchrony at the population level was a much better detector of the stimulus than the single afferent activity, as quantified by higher detectability values especially for lower durations (compare Figure 5D and Figure 3D, green; ANOVA with Bonferroni correction, *p* = 0.002, df = 295, *n* = 60). We quantified invariance (see section Materials and Methods) using both the single afferent activity as well as the synchrony measure and found significantly higher values for the latter (single neuron: mean: 0.08 ± 0.02 SD; max: 0.13; min: 0.04; synchrony: mean: 0.49 ± 0.06 SD; max: 0.58; min: 0.32; *p* = 7.791 × 10^–42^, *t*-test; Figure 5E). Similar results were observed when systematically varying the time scale at which synchrony was computed up to ∼60 ms (Figure 5G). Results obtained from simulations of our model at the population level were in good qualitative agreement with experimental data (Figures 5A–E, compare green and cyan throughout). Our model shows that the experimentally observed invariance of synchrony at the population level can be explained by the temporal filtering properties observed in electroreceptor afferents and further suggests that EA heterogeneities are not necessary to observe synchrony in EA pairs. Confirming this prediction, afferent heterogeneities as quantified by the geometric mean of the baseline firing rates of each pair did not affect invariance as no significant correlation was observed (Figure 5F, Pearson’ correlation coefficient, *r* = 0.1924; *p* = 0.2343). We conclude that synchronous activity at short timescales in receptor afferents displays invariance to variations in chirp duration.

**FIGURE 6:**
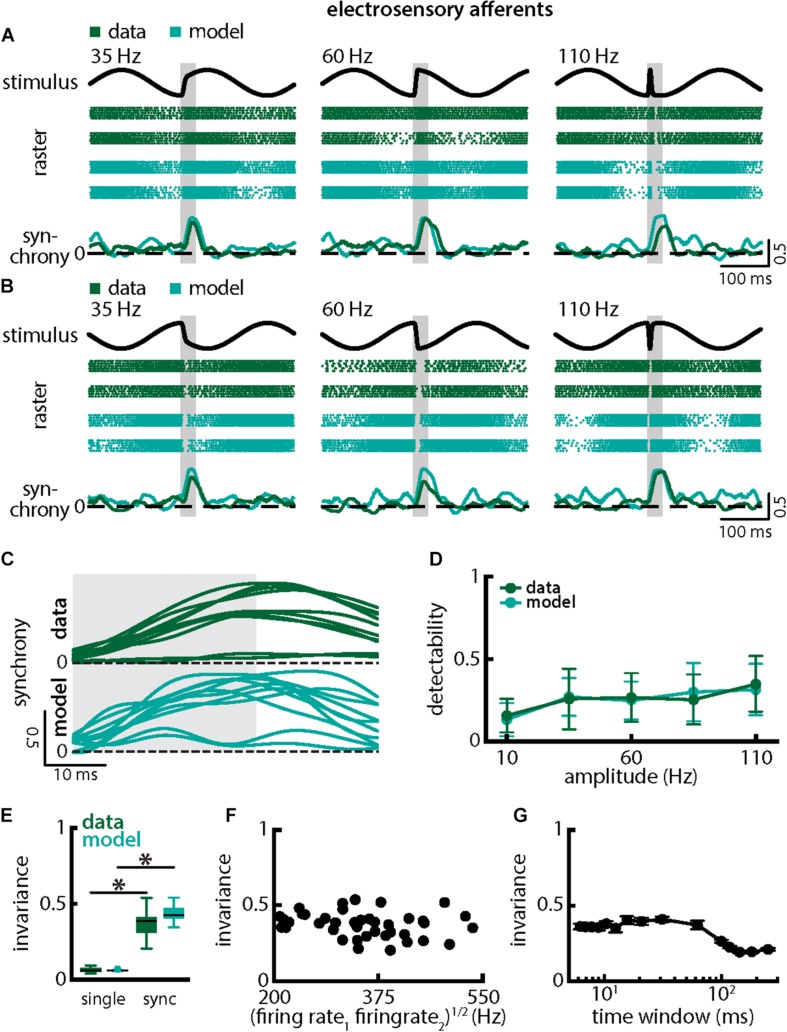
Synchrony provides an invariant representation of chirps with different amplitudes. **(A)** Example stimulus waveforms (top, black) for chirps with different amplitudes (left: 35 Hz; middle: 60 Hz; right: 110 Hz) occurring at the same phase of the 4 Hz beat, raster plots of two example afferents (middle top, green) and model neurons (middle bottom, cyan) showing responses to 5 out of 20 randomly chosen presentations (i.e., trials) of each stimulus and the time varying synchrony averaged over all 20 trials (bottom) from the shown example afferent (green) and model (cyan) pairs. The horizontal bars (shaded gray) represent the chirp window used for evaluation. The gray band shows the evaluation time window used to compute invariance (see below). **(B)** Same as **(A)**, but when the chirp occurred at a different phase of the beat. **(C)** Trial-averaged synchrony responses from the same example pair of afferents for experimental data (green) and from a pair of model afferents (cyan) for chirps of different amplitudes. **(D)** Population-averaged neuronal detectability values computed from the spiking synchrony from the afferents (green) and the model (cyan) as a function of amplitude. **(E)** Population-averaged invariance values computed for the afferents (green) and for the model (cyan) from single afferent activity (left) and from synchrony (right) for chirp amplitude. “^∗^” indicates statistical significance at the *p* = 0.05 level using a paired *t*-test. **(F)** Invariance as a function of the geometric mean of the afferent baseline firing rates for our dataset. No significant correlation was observed (Pearson’s correlation coefficient, *r* = –0.2148; *p* = 0.1832). **(G)** Invariance as a function of time window length. Invariance was more or less independent of time window length for values up to ∼60 ms.

Qualitatively similar results were obtained when investigating changes in chirp stimulus amplitude (Figure 6). The spiking activities of afferents were always more synchronized following the stimulus presentation (Figures 6A,B, green) thereby giving rise to similar increases in the synchrony measure (Figures 6A,B, bottom panels, green, 6C, top green). Stimulus detectability was also higher when considering synchrony than single neuron activity (Figure 6D, ANOVA with Bonferroni correction, *p* = 7.435 × 10^–9^, df = 295, *n* = 60). As chirps with different amplitude all gave rise to increases in synchrony with similar a timecourse (Figure 6C, top green), invariance was larger than when considering single neuron activity (Figure 6E, synchrony: mean: 0.49 ± 0.06 SD; max: 0.58; min: 0.32; amplitude: mean: 0.37 ± 0.07 SD; max: 0.54; min: 0.21; single neuron: mean: 0.06 ± 0.02 SD; max: 0.09; min: 0.04; *p* = 6.672 × 10^–30^, *t*-test). Similar results were observed when systematically varying the time scale at which synchrony was computed up to ∼60 ms (Figure 6G). Finally, results from modeling were in good qualitative agreement with experimental data (Figures 6A–E compare green and cyan throughout). Our model further confirms that the experimentally observed invariance of synchrony at the population level can be explained by the temporal filtering properties observed in electroreceptor afferents and further suggests that EA heterogeneities are not necessary to observe synchrony in EA pairs. Indeed, afferent heterogeneities as quantified by the geometric mean of the baseline firing rates of each pair did not affect invariance as no significant correlation was observed (Figure 6F, Pearson’s correlation coefficient, *r* = −0.2148; *p* = 0.1832). We conclude that synchronous activity at short timescales in receptor afferents displays invariance to variations in chirp amplitude.

### Single ELL Pyramidal Neuron Responses Decode Synchronous Afferent Activity as Their Responses Are More Invariant to Duration and Amplitude Than Those of Single Afferents

So far, our results have shown that, while single afferents respond differentially to natural electrocommunication stimuli with different durations and amplitudes, this is not the case when looking at the population level. This is because their activities are more synchronized irrespective of duration or amplitude, which leads to an invariant representation of electrocommunication stimuli. Information transmitted by neural activity is of course only useful if it is actually decoded by downstream neurons. As such, we next investigated the responses of ELL PCells that receive input from afferents to natural electrocommunication stimuli with different durations and amplitudes. PCells can be classified as either ON or OFF-type based on whether they respond to increased stimulation with increases or decreases in firing rate, respectively (Saunders and Bastian, 1984).

**FIGURE 7:**
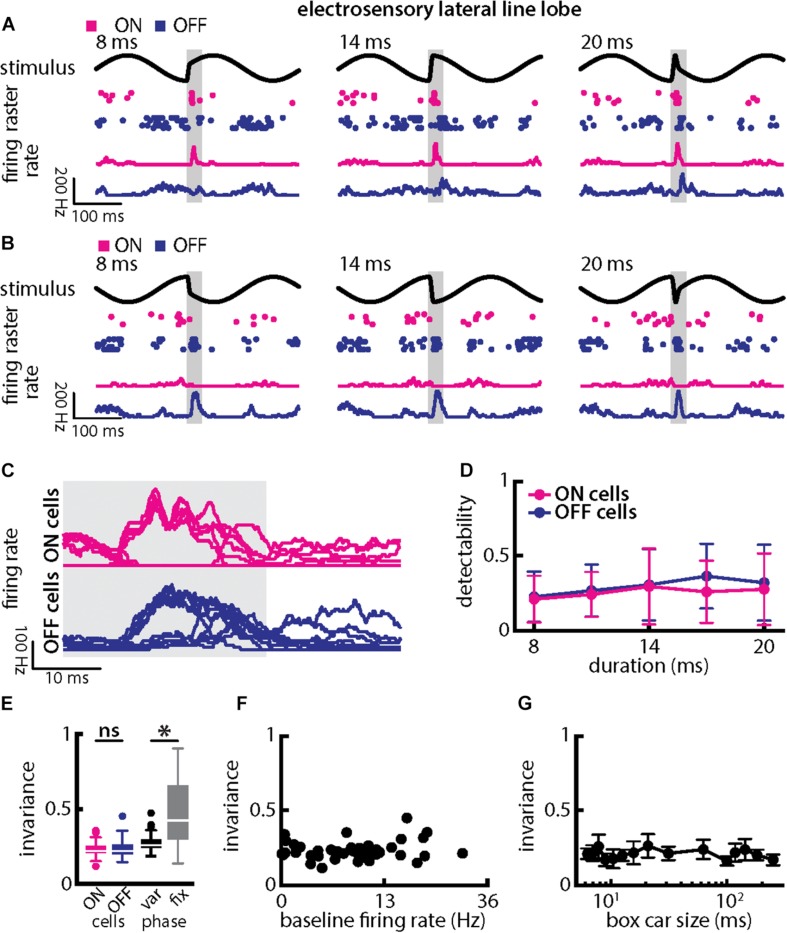
Central electrosensory neurons display more invariant representation of chirps with varying duration than peripheral electroreceptor afferents. **(A)** Example stimulus waveforms (top, black) for chirps with different durations (left: 8 ms; middle: 14 ms; right: 20 ms) occurring at the same phase of the 4 Hz beat, raster plots of an example ON-PCell (middle top, magenta) and OFF-PCell (middle bottom, blue) showing responses to 5 out of 20 randomly chosen presentations (i.e., trials) of each stimulus and corresponding firing rates of the same PCells averaged over all 20 trials (bottom). The gray band shows the evaluation time window used to compute invariance (see below). **(B)** Same as **(A)**, but when the chirp occurred at a different phase of the beat. **(C)** Superimposed trial-averaged firing rate responses of the same example ON (top panel) and OFF-type (bottom panel) PCells to chirps with different durations. **(D)** Averaged detectability values computed from firing rate responses for our ON-type PCell population (magenta) and our OFF-type PCell population (blue) as a function of duration. **(E)** Left: Population-averaged invariance values computed from ON (magenta) and OFF-type (blue) PCells for duration. Right: Invariance computed for all chirp phases used (left) and when only the phase that elicited excitatory responses in our PCell population was used (right) for varying chirp duration values. “^∗^” indicates statistical significance at the *p* = 0.05 level using a paired *t*-test. **(F)** Invariance as a function of baseline firing rate. No significant correlation was observed (Pearson’s correlation coefficient, *r* = 0.1505; *p* = 0.3539). **(G)** Population-averaged invariance as a function of the boxcar size used to obtain the time dependent firing rate from spiking activity.

When varying chirp duration (Figure 7), we found that single ELL PCells responded similarly to stimuli occurring at a given background beat phase (Figures 7A,B). Specifically, ON-type cells responded with increases in firing rate that were largely independent of chirp duration when the stimulus occurred at the beat trough (Figure 7A, magenta). In contrast, OFF-type cells responded with decreases in firing rate that were largely independent of duration for these stimuli (Figure 7A, blue). When the chirp stimulus instead occurred at the beat peak, the situation was reversed as ON-type cells responded with decreases in firing rate (Figure 7B, magenta) while OFF-type cells responded instead with increases in firing rate (Figure 7B, blue) that were in both cases largely independent of chirp duration. This is best seen by superimposing the different responses (Figure 7C). Stimulus detectability computed from ELL PCell activity was qualitatively similar for ON- and OFF-type cells (Figure 7D). This detectability was furthermore similar to that computed from afferent synchrony and thus significantly higher than that computed from single afferent activity (ON-type: *p* = 5.456 × 10^–17^; OFF-type: *p* = 2.046 × 10^–13^; ANOVA with Bonferroni correction). As such, invariance values among ON- and OFF-type ELL PCells were similar (Figure 7E, left; *p* = 0.525, *t*-test) but larger than those for single afferents (compare with Figure 5E; ON-type: *p* = 5.456 × 10^–17^; OFF-type: *p* = 2.046 × 10^–13^; ANOVA with Bonferroni correction). It should be noted that invariance values computed from single ELL PCells were lower owing to the fact that each cell type responded differentially when the stimulus occurred on different beat phases (compare Figures 7A–C). Invariance scores computed for a given phase were significantly higher (Figure 7E, right; *p* = 8.380 × 10^–8^; paired *t*-tests), which further confirms that single ELL PCell responses are more invariant than those of single afferents. It is important to note that the higher invariance scores seen for ELL PCells to chirps with different durations as compared to afferents is thus primarily due to the fact that trial-averaged firing rate responses were more similar to one another rather than variability. This is because the invariance measure is computed using the trial-averaged time dependent firing rates (i.e., firing rates averaged over repeated presentations of the stimulus waveform associated with a chirp with given duration and amplitude) rather than single-trial responses which are more variable (see section Materials and Methods). Pyramidal cell heterogeneities as quantified by the baseline firing rate did not affect invariance as no significant correlation was observed (Figure 7F; Pearson’s correlation coefficient, *r* = 0.1505; *p* = 0.3539). Invariance scores were furthermore robust to changes in the filter settings used to obtain the time-dependent firing rate from spiking activity (Figure 7G). Further, we note that previous studies have shown that some midbrain neurons receive balanced input from ON- and OFF-type ELL PCells (McGillivray et al., 2012; Aumentado-Armstrong et al., 2015) whose responses would then be expected to be more invariant as seen previously for other chirp attributes (Metzen et al., 2016).

**FIGURE 8:**
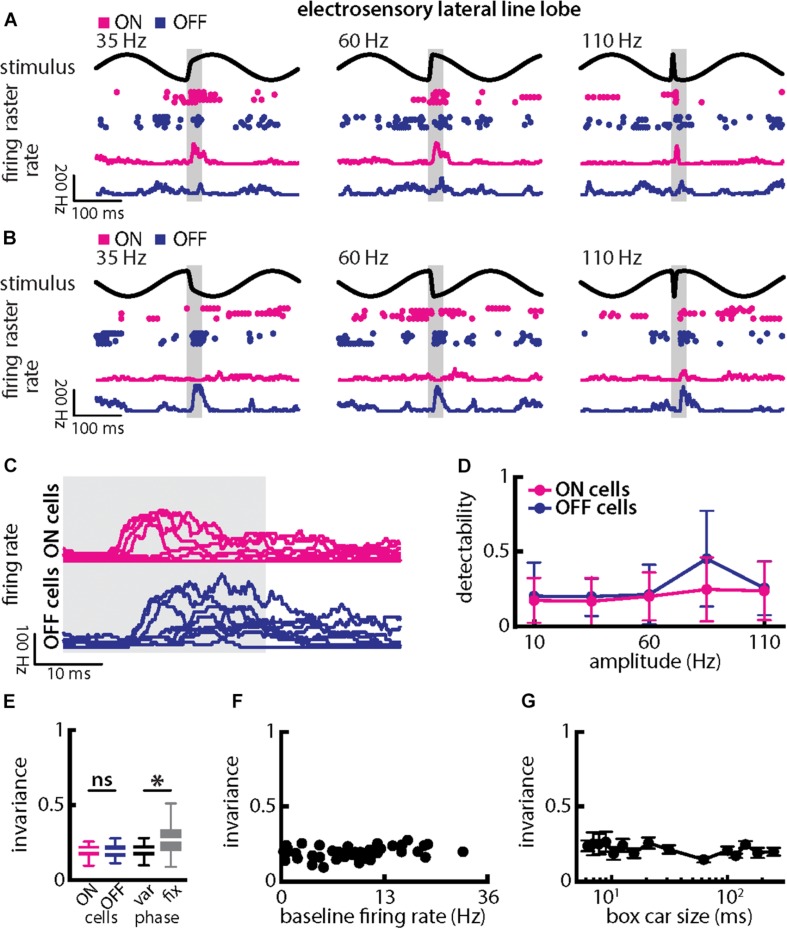
Central electrosensory neurons display more invariant representation of chirps with varying amplitude than peripheral electroreceptor afferents. **(A)** Example stimulus waveforms (top) for chirps with different amplitude (left: 35 Hz; middle: 60 Hz; right: 110 Hz) occurring at the same phase of the 4 Hz beat, raster plots of an example ON-PCell (middle top, magenta) and OFF-PCell (middle bottom, blue) showing responses to 5 out of 20 randomly chosen presentations (i.e., trials) of each stimulus and corresponding firing rates the same PCells averaged over all 20 trials (bottom). The gray band shows the evaluation time window used to compute invariance (see below). **(B)** Same as **(A)**, but when the chirp occurred at a different phase of the beat. **(C)** Superimposed trial-averaged firing rate responses of the same example ON (top panel) and OFF-type (bottom panel) PCells to chirps with different amplitudes. **(D)** Population-averaged detectability values computed from firing rate responses for our ON-type PCell population (magenta) and our OFF-type PCell population (blue) as a function of amplitude. **(E)** Left: Population-averaged invariance values computed from ON (magenta) and OFF-type (blue) PCells for amplitude. Right: Invariance computed for all chirp phases used (left) and when only the phase that elicited excitatory responses in our PCell population was used (right) for varying chirp amplitude values. “^∗^” indicates statistical significance at the *p* = 0.05 level using a paired *t*-test. **(F)** Invariance as a function of baseline firing rate. No significant correlation was observed (Pearson’s correlation coefficient, *r* = 0.2619; *p* = 0.1026). **(G)** Population-averaged invariance as a function of the boxcar size used to obtain the time dependent firing rate from spiking activity.

Qualitatively similar results were obtained when varying chirp amplitude. Overall, responses of ON- and OFF-type were largely independent of chirp amplitude when the stimulus occurred at a given background beat phase (Figures 8A–C, magenta and blue). Stimulus detectability was higher than that of single afferents (Figure 8D, ON-type: *p* = 2.686 × 10^–20^; OFF-type: *p* = 1.28 × 10^–20^; ANOVA with Bonferroni correction). As such, invariance values, although similar for ON- and OFF-type cells (Figure 8D, left; *p* = 0.954, *t*-test), were significantly higher than those obtained for single afferents (compare with Figure 6E; ON-type: *p* = 2.686 × 10^–20^; EAs vs. OFF-type: *p* = 1.28 × 10^–20^; ANOVA with Bonferroni correction). Invariance scores were lower owing to the fact that each cell type responded differentially when the stimulus occurred on different beat phases. Invariance scores computed for a given phase were significantly higher than those computed across phases (Figure 8E, right; *p* = 8.380 × 10^–8^; paired *t*-tests), owing to the fact that ON- and OFF-type cells responded differentially when stimuli occurred at different phases of the background beat (compare Figures 8A–C). Pyramidal cell heterogeneities as quantified by the baseline firing rate did not affect invariance as no significant correlation was observed (Figure 8F, Pearson’s correlation coefficient, *r* = 0.2619; *p* = 0.1026). Invariance scores were robust to changes in the filter settings used to obtain the time-dependent firing rate from spiking activity (Figure 8G).

Overall, our results strongly suggest that single ELL PCells decode synchronous afferent activity elicited by natural electrocommunication stimuli with different durations and amplitudes. This is because their response detectability and invariance are more consistent with those obtained from afferent synchrony than those obtained from single afferent activity.

### Weakly Electric Fish Display Behavioral Responses That Are Invariant to Natural Electrocommunication Stimuli With Varying Duration and Amplitude

**FIGURE 9:**
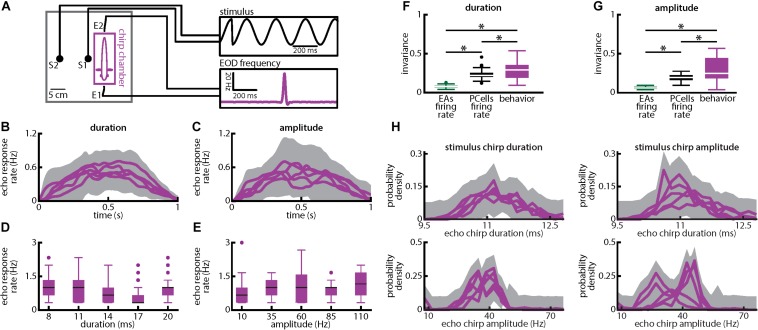
Weakly electric fish display invariant behavioral responses to chirps with varying duration and amplitude. **(A)** Experimental setup. Each fish (*N* = 8) was placed in an enclosure within a tank (chirp chamber). Stimuli were applied via two electrodes (S1 and S2) perpendicular to the fish’s rostro-caudal axis. The fish’s EOD frequency was recorded by a pair of electrodes positioned at the head and tail of the animal (E1 and E2). Behavioral responses consisted of communication stimuli characterized by transient increases in EOD frequency in response to the presented stimulus. **(B,C)** Population-averaged time dependent echo response rates for chirps of different durations **(B)** and amplitudes **(C)**. The shaded gray bands represent the 95% confidence intervals. **(D,E)** Population-averaged behavioral echo response rate (purple) for different durations **(D)** and amplitudes **(E)**. **(F,G)** Population-averaged invariance scores computed from behavioral responses (purple) in comparison to the neuronal invariance scores using single afferents (green) and PCells (black) obtained for different durations **(F)** and amplitudes **(G)**. “^∗^” indicates statistical significance at the *p* = 0.05 level using a one-way ANOVA with Bonferroni correction. **(H)** *Top:* Probability distributions of echo response duration for stimulus chirps of different durations (left) and amplitudes (right). *Bottom:* Probability distributions of echo response amplitude for stimulus chirps of different durations (left) and amplitudes (right). In all four cases, the probability distributions were not significantly different from one another (KS tests, *p* ≥ 0.1161 in all cases).

Finally, we investigated behavioral responses to chirps with different amplitudes and duration (Figure 9). To do so, we took advantage of the fact that *A. leptorhynchus* display “chirp echo responses” when stimulated with chirps (Hupé and Lewis, 2008) (Figure 9A; see Materials and Methods). Our results show that the behavioral responses elicited by chirp stimuli with different durations (Figure 9B) or amplitudes (Figure 9C) were similar to one another and that echo response rates were similar across different chirp durations (Figure 9D) as well as different chirp amplitudes (Figure 9E). Consequently, invariance values computed from behavioral responses were significantly higher than those obtained for either single afferents or PCells (Figure 9F; duration: EAs: *p* = 1.739 × 10^–15^; PCells: *p* = 0.033; Figure 9G; amplitude: EAs: *p* = 1.028 × 10^–15^; PCells: *p* = 5.125 × 10^–5^; ANOVA with Bonferroni correction). It is important to note that the behavioral responses (i.e., echo response rates) were most likely elicited by the chirp stimuli rather than the beat (see section Materials and Methods). It should furthermore be noted that differences in the timecourse of echo response rates that were most likely due to estimation error and/or fluctuations actually limited behavioral invariance values obtained here. These should thus be seen as lower bounds as is further discussed below. Our results thus show that behavioral responses were invariant to both chirp duration and amplitude, consistent with the hypothesis that changes in synchronous afferent activity, rather than changes in the single afferent firing rate, are decoded by ELL PCells. Our results have thus revealed that neural synchrony can be used to generate a neuronal representation that is invariant to stimuli with different attributes and how this representation is further processed downstream to presumably give rise to behavior. Moreover, the duration and amplitude of the echo response chirps elicited by the fish did not significantly change for different chirp parameters (Figure 9H; KS tests, p ≥ 0.1161 in all cases).

## Discussion

### Summary of Results

Here we investigated how electrosensory neural populations encoded natural electrocommunication stimuli with varying attributes (i.e., duration and amplitude) in order to mediate behavior. Despite the fact that both attributes were narrowly distributed under natural conditions, recordings from peripheral afferents revealed that, while single neurons encoded the different stimulus waveforms associated with different durations or amplitudes, all waveforms gave rise to increased synchrony either through excitation or inhibition at the population level. A phenomenological mathematical model reproduced experimental data showing that afferent responses at both the single neuron and population levels could be accounted for by single neuron filtering and spiking properties. Recordings from downstream central electrosensory neurons (i.e., ELL PCells) revealed that they decode information carried by synchronous activities of afferents as their responses were more invariant than those of single afferents. Specifically, ON-type cells were excited when afferents are excited synchronously while OFF-type cells were instead excited when afferents are inhibited synchronously. It is likely that ELL PCell responses are further processed by downstream brain areas to give rise to the observed invariant behavioral responses to natural electrocommunication stimuli. Our results thus reveal that neural synchrony can be used to generate an invariant representation to natural electrocommunication stimuli with different attributes as well as the mechanisms by which this representation is decoded by downstream neurons to presumably lead to behavioral responses at the organismal level.

### Feature Invariant Representations of Natural Electrocommunication Stimuli: Functional Consequences for Coding and Perception

The results of the current study have shown that electrosensory pathway encodes natural electrocommunication stimuli with different attributes. These are unlike those considered previously in which a natural electrocommunication stimulus with given attributes (i.e., the same amplitude and duration) occurred at different phases of the underlying background signal (Metzen et al., 2016; Metzen and Chacron, 2017), as seen under natural conditions (Walz et al., 2013; Aumentado-Armstrong et al., 2015). While single afferents encoded the resulting different stimulus waveforms differentially, synchrony between afferents at the population level provided an invariant representation that is decoded by downstream neurons to give rise to behavior (Metzen et al., 2016). Such invariant responses are desirable from a functional point of view because the probability at which natural electrocommunication signals occur is independent of the phase of the background signal at the time of emission (Walz et al., 2013; Aumentado-Armstrong et al., 2015). As such, these responses enable the organism to correctly perceive that different waveforms are actually generated due to the same electrocommunication signal (i.e., with given duration and amplitude).

As such, our result showing that natural electrocommunication stimuli with different amplitudes and durations are encoded in an invariant fashion by the electrosensory pathway is surprising. This is because, unlike the background beat phase considered above, both chirp amplitude and duration are instead narrowly distributed under natural conditions. Indeed, previous studies have shown that the natural electrocommunication signals differ in terms of duration and amplitude across different Apteronotid species and could thus be used in theory to distinguish between con- and hetero-specifics (Petzold et al., 2016). This is even more surprising because we considered chirps with attributes that were well outside of the range observed for chirps emitted by fish (see Figure 2). However, our results show that such “un-natural” chirps gave rise to neural (in terms of EA synchrony) and behavioral responses that closely resembled those observed for more “natural” chirps. Our results thus provide evidence against (but do not disprove, see below) the hypothesis that differences in chirp duration and amplitude are encoded by the electrosensory system and can be perceived by the organism. Specifically, they suggest that despite large differences in their attributes, such stimuli are all ultimately perceived similarly. If correct, then this hypothesis greatly complicates the problem of distinguishing between conspecific and heterospecific individuals based on chirp characteristics. Our results support the proposal that the functional role of chirps is to temporarily suppress electrosensory neuronal responses to other stimuli (i.e., temporarily “blind” the opponent) (Hupé and Lewis, 2008). This is because peripheral afferent activities will then be synchronized irrespective of stimulus attributes. Further evidence for this hypothesis comes from previous electrophysiological studies showing that both ELL (Marsat et al., 2009; Vonderschen and Chacron, 2011) and TS (Vonderschen and Chacron, 2011) neurons are best at detecting the presence of natural electrocommunication stimuli rather than at discriminating between differences in stimulus attributes.

That said, it is important to note that our results do not imply that weakly electric fish cannot distinguish between chirps with different attributes. Specifically, our results do not rule out the possibility that the animals can actually perceive differences in chirp amplitude and duration but simply do not report them behaviorally. Indeed, it is possible that ELL pyramidal cells other than the ones considered here (i.e., in other segments) could actually decode information about stimulus attributes carried by single peripheral afferents. This possibility is however unlikely because previous studies have shown that the ELL pyramidal cells within the lateral segment considered here give the strongest responses to natural electrocommunication stimuli (Marsat et al., 2009). It is furthermore important to note that previous studies have shown that the invariant neuronal responses due to synchrony and the invariant behavioral responses with given attributes occurring at different phases of the underlying background both deteriorate when higher beat frequencies are considered (Metzen and Chacron, 2017). This is because EA synchrony during the chirp is much weaker for higher beat frequencies and thus more commensurate with that seen during the beat (Walz et al., 2014). Importantly, our previous results showing that both neural and behavioral invariance deteriorate for higher beat frequencies provides a strong link between changes in invariance due to EA synchrony and changes in behavioral invariance (Metzen and Chacron, 2017). Under natural conditions, the beat frequency can reach much higher values (e.g., 400 Hz) than the one considered in the current study and we predict that, as seen for phase-invariance, both the duration and amplitude-invariant neural and behavioral responses seen here would deteriorate when higher beat frequencies are used. Future studies should thus investigate how increasing the beat frequency affects invariant coding and perception of electrocommunication stimuli with different durations and amplitudes.

We further hypothesize that the invariant neuronal and behavioral responses to natural electrocommunication stimuli considered here would break down when the stimulus contrast is increased beyond that explored in this study which is experienced when fish are located ∼13 cm from one another (Yu et al., 2019). Indeed, higher contrasts are experienced when two conspecifics move closer (i.e., within 5 cm) to one another (Yu et al., 2012, 2019), or when the beat frequency is increased. This is because we predict that peripheral afferents will then display stronger phase locking (i.e., only fire during specifics phases) to the background signal, which will increase their synchrony. In the case of increasing beat frequency, this is due to their known high-pass tuning characteristics (Xu et al., 1996; Metzen and Chacron, 2017). As such, we propose that weakly electric fish will be able to discriminate between natural electrocommunication stimuli with different attributes whenever these are produced when both animals are when in close proximity to another or during high frequency beats. Further studies are needed to test this hypothesis. If true, our hypothesis would provide an explanation as to recent field results showing that natural electrocommunication signals are sometimes produced when both animals are located close to one another or during high frequency beats (Henninger et al., 2018).

Our results have shown that invariant responses of EA synchrony to chirps with varying amplitude and duration are likely decoded by ELL PCells to presumable lead to behavior. However, it should be noted that our study only considered synchrony between EA pairs whereas the PCells within the lateral segment considered here receive input from ∼1000 EAs (Maler, 2009). Previous studies have shown that PCells display ion channels such as persistent sodium which would favor detection of coincident EA activity (Noonan et al., 2003). Further, modeling studies have suggested that the tuning properties of PCells within the lateral segment emerge because they actually detect coincident EA input (Middleton et al., 2009). However, integration of EA input by PCells within the lateral segment has not been systematically studied experimentally. For example, the so-called “synchrony receptive fields” (Brette, 2012) (e.g., the fraction of EA firing synchronously needed to elicit PCell firing, or the time window during which EA activity can be considered synchronous) remain unknown to date. While previous results (Marsat et al., 2009; Marsat and Maler, 2010; Metzen et al., 2016; Metzen and Chacron, 2017) and the results of the current study are consistent with the hypothesis that PCells within the lateral segment detect coincident EA activity, further studies are needed to fully test this hypothesis.

Further, we note that our behavioral invariance values were actually lower than those obtained for EA synchrony. As mentioned above, this is likely due to the fact that the former were limited by fluctuations and we predict that behavioral invariance values are actually higher. Further studies are however needed to understand how the activities of PCell population are integrated downstream. Previous studies have shown that some midbrain electrosensory neurons display invariant responses to beat phase (i.e., a neural correlated of the observed behavioral invariance to beat phase) by integrating input from ON- and OFF-type cells (Metzen et al., 2016). We hypothesize that this mechanism will give rise to responses in midbrain neurons that are fully invariant to chirps of different amplitudes or durations irrespective of the beat phase at which they occur. While there is anecdotal evidence that such neurons exist (see Figures 2C, 8A of Vonderschen and Chacron, 2011), the responses of midbrain neurons to stimulation protocols similar to the ones used in the current study have not been systematically investigated to date and should be the focus of future studies.

It is important to note here that both EAs and ELL PCells display significant heterogeneities in terms of baseline activity as well as responses to stimuli (Bastian, 1981; Bastian and Nguyenkim, 2001; Bastian et al., 2002, 2004; Gussin et al., 2007; Savard et al., 2011). While it is clear that heterogeneous populations are advantageous for coding (Stocks, 2000; Padmanabhan and Urban, 2010; Brette, 2012; Mejias and Longtin, 2012), our modeling and experimental data suggest that heterogeneities are not necessary to observe the phenomena described in the current study. Specifically, our modeling, which was based on a homogeneous neural population, reproduced our experimental data both at the single neuron and population levels for EAs. Moreover, we found no significant correlation between invariance and the baseline firing rate, which is strongly correlated with morphological differences in ELL PCells (Bastian and Nguyenkim, 2001; Bastian et al., 2004). Further studies are needed in order to investigate the effects of neural heterogeneities on invariance coding at both the EA and ELL PCell level. For the former, these should investigate how EA heterogeneities influence the so-called “synchrony receptive fields” of ELL PCells mentioned above. For the latter, the effects of PCell heterogeneities should also be more systematically investigated. This is particularly important as previous studies have shown that a strong factor contributing to PCell heterogeneities is the amount of descending input (i.e., feedback) that is received from higher brain centers. The effect of such feedback has been mostly studied at the single neuron level (Bastian, 1986; Chacron et al., 2005b; Marsat and Maler, 2012; Huang et al., 2018, 2019; Metzen et al., 2018) and further studies are needed to understand whether and, if so, how such feedback can facilitate detection of EA synchrony by ELL PCells.

Finally, we note that the electrocommunication stimuli considered in the present study primarily occur during agonistic encounters and, as such, correspond to the “type II chirps” described previously. It is important to note that *A. leptorhynchus* emit other types of natural communication stimuli that are not considered here (Zakon et al., 2002). In particular, they tend to emit another type of communication signal termed “type I chirps” during mating behavior. Electrophysiological studies have shown that neuronal responses to these are fundamentally different (Marsat and Maler, 2010; Vonderschen and Chacron, 2011; Allen and Marsat, 2018). Future studies are needed to investigate how electrosensory neuronal populations encode other natural electrocommunication signals not considered here. In particular, it will be important to consider the fact that ELL PCell trial-to-trial variabilities to repeated stimulus presentations are correlated (Chacron and Bastian, 2008; Simmonds and Chacron, 2015; Hofmann and Chacron, 2018), which has been ignored by previous studies (Marsat et al., 2009; Marsat and Maler, 2010; Allen and Marsat, 2018). Such “noise” correlations can have profound influence on coding by neuronal populations (Averbeck et al., 2006; Cohen and Kohn, 2011; Doiron et al., 2016; Franke et al., 2016; Zylberberg et al., 2016) and are likely to be found in all ELL PCells as they are due to shared input from peripheral afferents (Hofmann and Chacron, 2017, 2018).

### Implications for Other Systems

Here we have provided the first experimental evidence that synchrony can enable the emergence of a neuronal representation that is invariant to stimuli with different attributes such as amplitude and duration. Such invariant representations are also seen in other systems (auditory: Bendor and Wang, 2005; visual: Zoccolan et al., 2007; olfactory: Martelli et al., 2013). In all cases, tolerance to variations in identity-preserving transformations such as size, contrast, or viewpoint progressively increases in neurons at higher processing stages (Dicarlo and Cox, 2007). The mechanisms leading to such an increase in invariance are not fully understood to date. Our results showing how neural synchrony, which is observed ubiquitously in the central nervous system (Uhlhaas et al., 2009; Harris and Gordon, 2015), gives rise to a neuronal representation that is invariant to both amplitude and duration is thus likely to be shared by other systems/species. This is because invariance to stimulus amplitude has been observed in the visual (Anderson et al., 2000), auditory (Billimoria et al., 2008; Barbour, 2011), somatosensory (Pei et al., 2010), and olfactory (Storace and Cohen, 2017) systems. The fact that the electrosensory system studied here displays both anatomical and functional similarities with other systems (Clarke et al., 2015) suggests that neural synchrony also plays a role in mediating the emergence and refinement of such representations in other systems. Further studies are however needed to test this prediction.

## Data Availability Statement

The datasets (analyzed) and codes for this study can be found in the figshare repository (10.6084/m9.figshare.8041136).

## Ethics Statement

The animal study was reviewed and approved by McGill University’s animal care committee under protocol number 5285.

## Author Contributions

MM, VH, and MC: conceptualization, funding acquisition, methodology, validation, writing, review, and editing. MM: data curation, formal analysis, visualization, and writing – original draft. MM and VH: investigation. MC: project administration, resources, software, and supervision.

## Conflict of Interest

The authors declare that the research was conducted in the absence of any commercial or financial relationships that could be construed as a potential conflict of interest.
